# Infrared Drying as a Quick Preparation Method for Dried Tangerine Peel

**DOI:** 10.1155/2017/6254793

**Published:** 2017-11-19

**Authors:** Mingyue Xu, Guifang Tian, Chengying Zhao, Aftab Ahmad, Huijuan Zhang, Jinfeng Bi, Hang Xiao, Jinkai Zheng

**Affiliations:** ^1^Institute of Food Science and Technology, Chinese Academy of Agricultural Sciences, Beijing 100193, China; ^2^State Key Laboratory of Chemical Resource Engineering, Beijing University of Chemical Technology, Beijing 100029, China; ^3^Department of Food Science, University of Massachusetts, Amherst, MA 01003, USA; ^4^College of Bioscience and Biotechnology, Hunan Agricultural University, Changsha, Hunan 410128, China

## Abstract

To establish the most convenient and effective method to dry tangerine peels, different methods (sun drying, hot-air drying, freeze drying, vacuum drying, and medium- and short-wave infrared drying) were exploited. Our results indicated that medium- and short-wave infrared drying was the best method to preserve nutraceutical components; for example, vitamin C was raised to 6.77 mg/g (D.W.) from 3.39 mg/g (sun drying). Moreover, the drying time can be shortened above 96% compared with sun drying. Importantly, the efficiency of DPPH radical scavenging was enhanced from 26.66% to 55.92%. These findings would provide a reliable and time-saving methodology to produce high-quality dried tangerine peels.

## 1. Introduction

Tangerine (mandarin) is the second most important citrus genera, whose specific species is* Citrus reticulata* [1]. The data from the Food and Agriculture Organization (FAO) of the United Nations showed that China is the largest producer of tangerines in the world that produced 15.17 million tons of tangerines in 2013. Tangerine is usually consumed as a fresh fruit in China because of its delicious taste and high nutritional components. In addition, tangerines were mostly processed to produce juice and canned tangerines [2]. Tangerine peels are usually treated as agroindustrial waste. However, they are rich in various nutraceutical components, including essential oils, flavonoids, pectin, and carotenes [2, 3]. Currently, the applications of these functional components are mainly focused on cosmetics, foods, dyes, and medicines [3–5].

It is also well known that tangerine peels are the main raw material to produce dried tangerine peel that can be used as an ingredient in traditional Chinese medicine as well as functional foods. Dried tangerine peel could be produced by drying fresh peels of* Citrus reticulata* Blanco or its cultivars collected between September and December in sun or at low temperatures [6]. It has been reported to alleviate indigestion, improve cardiac circulation, and suppress inflammatory syndromes of the respiratory tract such as bronchitis and asthma [7, 8]. Fresh tangerine peels were reported to have less biological functions than dried tangerine peels. The relatively lower biological importance of fresh tangerine peels is correlated to its high water content [9] and the low concentration of the function compounds, such as hesperidin. The Chinese pharmacopoeia explicitly stipulated that moisture content in Pericarpium Citri Reticulatae must be no more than 13% and that hesperidin content in Pericarpium Citri Reticulatae must be more than 3.5%. Hesperidin, an abundant and medicinally important bioflavonoid in tangerine peels, which determines the quality of dried tangerine peel, plays a vital role in preventing tumors, diminishing inflammation, performing bacteriostasis, and lowering cholesterol levels [10]. Literature survey indicates that phenolic compounds, especially flavonoids, are an important class of bioactive phytochemicals that provides many health benefits, such as hypocholesterolemic, hypoglycaemic, antioxidant, anti-inflammatory, anticancer, and antiatherogenic activities [7]. Synephrine, another essentially functional component in dried tangerine peel, is used to dilate blood vessels, decrease blood pressure, and expand trachea and bronchus [5]. Similarly, vitamin C, a vital nutrient found in citrus, can promote body growth and enhance its resistance to disease. It also plays an important role in preventing and curing scurvy, especially in the early stages [11]. With all these beneficial bioactive compounds found in Pericarpium Citri Reticulatae, it is important to investigate the variation of these functional components during the drying of tangerine peels.

The main preparation method of dried tangerine peel, sun drying, has the advantages of low cost and simple operations. However, the quality of tangerine peels dried in the sun was inhomogeneous and unstable because it was very susceptible to weather and environments variations, such as natural disasters, insects, and dust [12]. Moreover, the sun drying method is very time-consuming to produce dried tangerine peel, making it vital to seek new drying methods for production. Nowadays, many drying methods had been used to dry fruits and vegetables, such as hot-air drying for grape leather, freezing drying for button mushroom, vacuum drying for carrot slices, and infrared drying for potato slices [13–15].

In spite of previous studies on drying processes of tangerine peels [12, 16, 17], there is no detailed report on the influence of different drying methods on the nutraceutical components in tangerine peels, which are important indexes for assessing the quality of dried tangerine peel. This study systematically investigated the influence of different drying methods (sun drying, hot-air drying, freeze drying, vacuum drying, and medium- and short-wave infrared drying) on the main compositions of tangerine peels (moisture, soluble solid, reducing sugars, total sugar, crude fiber, titratable acid, ash content, and minerals), major functional components (total phenolic compounds, total flavonoids, hesperidin, synephrine, and vitamin C), and the antioxidant activity of the dried tangerine peels.

## 2. Materials and Methods

### 2.1. Chemicals

Folin & Ciocalteu's phenol reagent, 1,1-diphenyl-2-picrylhydrazyl radical (DPPH), 2,4,6-tripyridyl-1,3,5-triazine (TPTZ), 2,2′-azinobis-(3-ethylbenzthiazoline-6-sulfonic acid) (ABTS), 6-hydroxy-2,5,7,8-tetramethylchroman-2-carboxylic acid (Trolox), gallic acid monohydrate, 2,6-dichloroindophenol, (+)-catechin hydrate, and vitamin C were purchased from Sigma-Aldrich (Shanghai, China). The hesperidin was purchased by Chengdu Institute of Biology, Chinese Academy of Sciences (Chengdu, China). The synephrine was purchased from National Institute for the Control of Pharmaceutical and Biological Products (NICPBP, Beijing, China). All other reagents of HPLC-grade and analytical grade were purchased from Sinopharm Chemical Reagent Co., Ltd. (Beijing, China).

### 2.2. Material Pretreatment

Fresh tangerines (*Citrus reticulata* v. tangerine) were grown in Jiangmen city (Guangdong Province, China) and purchased (November 2014) from a local market in Beijing. The ripe tangerines, with an average weight of 151.83 ± 4.65 g, were washed, dried, cut into eight pieces, and peeled carefully by hand without destroying their shape. The initial moisture content of tangerine peels was 2.50 ± 0.10 g water/g dry material and the thickness of tangerine peels was 2.68 ± 0.11 mm measured with Vernier caliper.

### 2.3. Drying Processes and Conditions

The tangerine peels were dried by 5 methods, which were sun drying (SD), freeze drying (FD), hot-air drying (HAD), vacuum drying (VD), and medium- and short-wave infrared drying (IRD). The weight loss was recorded by a CPA-125 digital electronic balance purchased from Sartorius Instrument System Co., Ltd. (Beijng, China), with the precision of 0.1 mg. Tangerine peels were dried until the moisture content was less than 0.05 g water/g dry material. Thirty grams of tangerine peels was placed in a sample steel tray (30 × 50 cm^2^) with 60 holes whose diameter was 5 cm to form a layer of 0.5 cm before being dried. The process of each drying method was briefly described as follows.

(*1) SD*. The sample tray was exposed to sunlight from 9 am to 4 pm at the ambient temperature (15~20°C) with relative humidity within 30%–40%. The sample was taken out every 5 hours and weighed.

(*2) FD*. It was carried out in a freeze dryer (Alphal-4 Lplus, Marin Christ, Germany) at −30°C under 10^−3^ Mpa. The sample was taken out every 2 hours and weighed.

(*3) HAD*. HAD was performed in an oven (DHG-9070, Shanghai Yiheng Scientific Instruments Co., Ltd., China) at 60, 70, 80, and 90°C with a fixed air velocity of 2.1 m/s. Hot air was supplied in vertical model. The samples were taken out every 10 minutes and weighed.

(*4) VD*. The drying was carried out in vacuum dryer (D2F6090, Shanghai Jinghong Laboratory Instrument Co., Ltd., Shanghai, China) at 60, 70, 80, and 90°C with a fixed vacuum degree of 3 × 10^−3^ Mpa. The samples were taken out every 20 minutes and analyzed for weight loss.

(*5) IRD*. IR was conducted in a medium- and short-wave infrared radiation dryer (Senttech, Shengtaike Infrared Technology Instrument Co., Ltd., China). The drying was performed at different temperatures (60, 70, 80, and 90°C) with a power consumption of 1350 w and fixed air velocity of 2.1 m/s. The samples were taken out every 5 minutes and weighed.

### 2.4. Drying Kinetics

The drying kinetics of tangerine peels were based on weight loss, which was represented by drying curve and drying rate curve. Drying curve was presented as moisture ratio versus drying time and drying rate curve was presented as drying rate versus moisture content [17].

The initial water content is associated with a reduction in weight of tangerine peels before and after drying. The initial moisture content of tangerine peels was obtained by comparing the amount of evaporated water with drying samples at 105 ± 1°C until the weight was not changed. The moisture content at any time (*X*_*t*_) was shown in the following [12]:

(1) $$\begin{array}{c} X_{t}=\frac{W_{t}-W_{0}}{W_{0}}, \end{array}$$

where *W*_0_ is the dry weight of sample and *W*_*t*_ is the weight at any time.

The moisture ration (MR) was obtained by the following [12]:

(2) $$\begin{array}{c} MR=\frac{X_{t}-X_{f}}{X_{0}-X_{f}}, \end{array}$$

where *X*_0_ is the initial moisture content, *X*_*f*_ is the final moisture content (0.05 g water/g dry weight), and *X*_*t*_ is the moisture content at any time.

The drying rate (*R*_*i*_) was obtained by the following [17]:

(3) $$\begin{array}{c} R_{i}={\left(\;\frac{dX}{dt}\;\right)}_{i}. \end{array}$$

### 2.5. Main Components of Dried Tangerine Peels

The soluble solid was measured by using a digital-display refractometer (WZB 45, Shanghai Precision Scientific Instrument Co., Ltd., Shanghai, China) and the results were obtained by refractive index multiplied by the dilution ratio [18]. Total sugars, reducing sugars, crude fiber, ash content, and titratable acids were determined by the phenol-sulphuric acid method [19], DNS method [20], the gravimetric method using a fiber tester, the muffle furnace (Lindberg/Blue, Thermo Fisher, USA) at 550°C [17], and the potentiometric titration method [18], respectively.

### 2.6. Extraction

**Figure 1:**
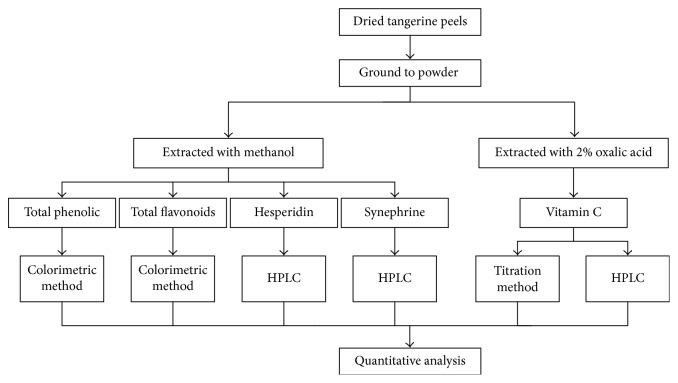
Flowchart of extracting of total phenolics, total flavonoids, hesperidin, synephrine, and vitamin C.

As shown in Figure 1, dried tangerine peels were grinded with a pestle in liquid nitrogen. One gram of sample was homogenized with 10 mL methanol for ultrasonic-assisted extraction for 1 h. Then, the mixture was centrifuged at 10,000 rpm for 10 min. The extraction procedure was repeated again. The supernatants were combined to detect total phenolic compounds, total flavonoids [21], hesperidin [22], and synephrine contents [5]. In order to analyze the vitamin C content, 1 g powder samples were extracted with 10 mL 2% oxalic acid and the mixture was centrifuged at 7600 rpm for 20 min to collect the supernatant [23]. Pure ethanol and 50% methanol were also used to optimize solvents for the extraction of hesperidin and synephrine.

### 2.7. Determination of Functional Components

#### 2.7.1. Total Phenolics Content and Total Flavonoids Content

Folin-Ciocalteu method and colorimetric assay were used to determine the total phenolic content and total flavonoids content of tangerine peels with some modification, respectively [21]. When the total phenolic content was measured, the optical density was measured at 765 nm using a UV spectrophotometer (UV-1800, Shimadzu, China). Gallic acid was used as a reference standard. The value of total phenolic content was expressed as mg gallic acid equivalents (GAE) per g dried sample (mg GAE g^−1^ d.w.). While the total flavonoids content was measured, the absorbance was 510 nm. The detection of total flavonoids content was based on the standard curve of (+)-catechin hydrate, which was expressed as mg (+)-catechin hydrate equivalents (CHE) per g dried sample weight (mg GHE g^−1^ d.w.).

#### 2.7.2. Hesperidin Content and Synephrine Content

HPLC was used to determine hesperidin and synephrine as previously designed methods with some modifications [5, 22]. The absorbance was 278 nm, the injection volume was 10 *μ*L and flow rate was maintained at 1 mL/min. The mobile phase system was comprised of methanol as mobile phase A and 0.1% (v/v) formic acid in deionized water as mobile phase B. The linear solvent gradient was composed of 30% A from the beginning to 5 min, 5% A at 15 min, 7% A at 20 min, and 30% A from 25 min to 30 min.

#### 2.7.3. Vitamin C Content

The vitamin C content was determined by two methods, namely, 2,6-dichloroindophenol titration method [24] and HPLC method [23]. 10 mL diluted supernatant was titrated against 2,6-dichloroindophenol solution. The process of titration was terminated when a pink color appeared and lasted for at least 15 s (end point). The blank experiment was performed with 10 mL 2% oxalic acid solution. The HPLC method for vitamin C determination was performed as follows. The mobile phase was composed of methanol as mobile phase A and phosphate buffer with 2% methanol as mobile phase B. The injection volume was 10 *μ*L, the flow rate was maintained at 1 mL/min, and absorbance was set up at 245 nm. The linear solvent gradient was composed of 100% B at the beginning, 20% B at 9 min, 20% B at 9.1 min, and 100% B from 14.1 min to 20 min.

### 2.8. Antioxidant Capacity Assays

One gram of dried tangerine peels was mixed with 20 mL MeOH and sonicated for 2 h. After being centrifuged for 10 min at 10000 r/min, the supernatant was obtained. This process is repeated and the supernatant was combined and stored in 4°C. Before determination, the supernatant was diluted 40 times with MeOH. Antioxidant capacity was evaluated by three assays: free radical scavenging activity by DPPH method, total antioxidant capacity by ABTS method, and ferric reducing ability by FRAP method [25]. The detection wavelengths were 517, 734, and 593 nm for DPPH, ABTS, and FRAP assays, respectively. Trolox was used as a standard. The results were expressed as the percentage of DPPH radical scavenged, the percentage of ABTS^∙+^ radical scavenged, and *μ*mol Trolox equivalent antioxidant capacity per 100 mg dried sample weight (*μ*mol TE/100 mg dw) for DPPH, ABTS, and FRAP assays, respectively.

### 2.9. Statistical Analysis

All measurements were performed in triplicate. The experimental data were analyzed by the SPSS 19.0. Differences between quality indexes affected by different treatments were determined by ANOVA procedure (*P* < 0.05) and least significant difference (LSD) method. The figures were plotted by the origin 8.0.

## 3. Results and Discussion

It is generally known that the dried tangerine peels have more potential for storage and application than the fresh ones. Moisture content was one of the major differences between the dehydrated and the fresh tangerine peels. But the drying process not only reduced the moisture content but also had great effect on the nutraceutical components, including main compositions and functional components of tangerine peels.

### 3.1. Effect of Drying Conditions on Drying Kinetics of Tangerine Peels

The tangerine peels were dried by sun drying (SD), hot-air drying (HAD), freeze drying (FD), vacuum drying (VD), and infrared drying (IRD), respectively. The drying time with respect to moisture ratio and moisture content with respect to drying rate were investigated to evaluate the effect of different drying methods, respectively.

**Figure 2:**
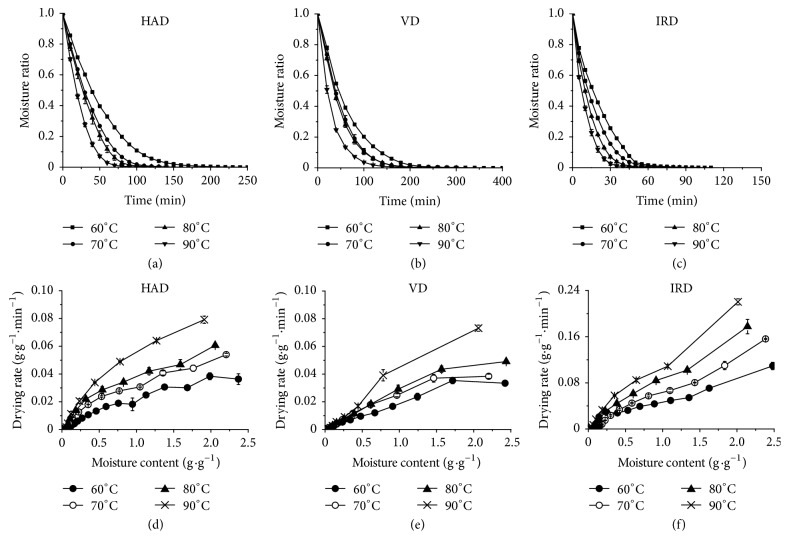
Drying curves and drying rate curves of tangerine peels dried by hot-air drying (a, d), vacuum drying (b, e), and medium- and short-wave infrared radiation drying (c, f).

The results of FD and SD were not shown in Figure 2 because samples dried by freeze drying and sunlight needed about 1320 min and 3000 min to reach the final moisture content, respectively. Figures 2(a), 2(b), and 2(c) showed that the drying times of tangerine peels at 60, 70, 80, and 90°C in HAD, VD, and IRD ranged from 150 to 200 min, 280 to 400 min, and 75 to 110 min, respectively. The drying time decreased with the increase of temperature. Among the tested drying methods, the SD was the most time-consuming method followed by FD, VD, and HAD. IRD was the fastest method to dry tangerine peels among all the methods. The longer drying time during the SD method may be related to a low and unstable temperature and the sample susceptibility to air humidity [26, 27]. Because the processes of freezing and sublimation are also time-consuming, FD takes longer time to dry a sample. The heat of HAD could be delivered by thermal convection in vertical supply model from outside into the tangerine peels. However, the radiant heat of the IRD might be directly generated deep inside the samples, which led to an efficient inside-out heat transfer. The moisture in VD remained for a longer time than HAD, so the drying time of VD was longer. In a vacuum oven, the moisture on the surface of samples evaporates quickly but the inside moisture cannot migrate to the surface in time; hence, a film is formed on the surface of samples, which impeded the inside moisture migration and prolonged drying time [14].

Figures 2(d), 2(e), and 2(f) illustrated that the falling-rate of drying period was the main drying process of tangerine peels, which was similar to the drying processes of sweet potato [28]. These results suggested that the drying rate was increased with the increase of temperatures. For example, when the moisture ratio of samples in hot-air dryer was decreased from 1 to 0.2, the drying times at 60, 70, 80, and 90°C were about 80, 70, 55, and 40 min, respectively. The phenomena were the same as VD and IRD. The decrease in drying time at elevated temperatures and low moisture content may be attributed to the increase in the temperature difference between the drying air and the product, which accelerated the speed of water diffusion [29].

### 3.2. Effect of Different Drying Methods on Main Compositions of Tangerine Peels

Table 1 showed the main components of tangerine peels dried at different methods, including soluble solid, reducing sugar, total sugar, crude fiber, titratable acid, ash content, and mineral content (Na, Mg, K, Ca, Fe, Mn, Cu, Zn, and Se). The contents of these components have a close relationship with the functions of dried tangerine peels. 80°C was set as the dried temperature of HAD, IRD, and VD because the main components of tangerine peels were high at 80°C of different drying methods and the dried time was long at low temperature.

Soluble solids in different dried tangerine peels were in the range of 41%~54%, while those in fresh tangerine peels were 14%. It is known that dried samples had higher soluble solids than the fresh ones. The tangerine peels had the most soluble solids after IRD and FD treatment, while the peels had the least soluble solids after SD treatment. The soluble solids are a relatively important index because dried peels were always soaked in water to drink. So IRD and FD were suitable ways to store soluble solids.

The sugar in tangerine peels might be the substrate of other compounds, such as vitamin C and glycoside. The investigation of sugar content is beneficial in providing the change mechanism of the other compounds. The reducing sugar content of tangerine peels after different drying processes was in the range of 489.43~545.59 mg/g dw, and the total sugar content of tangerine peels after different drying processes was in the range of 325.86~553.45 mg/g dw. The reducing sugar and total sugar content of the dried peels were both lower than those of fresh ones (725.41 mg/g dw, 753.50 mg/g dw). The sugars might be degraded while heating the samples for a long time during SD treatment. The samples dried by SD had the lowest total sugar content, while samples after FD had the highest one (553.45 mg/g dw).

The crude fiber was the major ingredient of cell walls. The change of its content could reflect the cell's membrane damage degree. The crude fiber content in fresh samples was 12.8%, while that in dried samples was lower in the range of 10.5%~11.1%, in line with previous report [21]. The results showed that the drying process had a slight effect on the cell wall damage.

The titratable acid content had a great effect on the flavor quality of dried tangerine peels. Compared with 0.875% in fresh peels, the titratable acid content in dried samples was in the range of 0.355%~0.835%. Of all drying methods, the titratable acid content was the highest after IRD and HAD treatment and was the lowest after SD treatment. The organic acids degrade much more rapidly during sun drying.

The storage and flavor quality of tangerine peels was also affected by the mineral content. When 80°C was set as the dried temperature of HAD, IRD and VD, the amounts of Na, Mg, Ca, Mn and Zn in fresh samples were higher than those in dried samples. Moreover, the amounts of Fe and Se in fresh ones were higher than those in dried samples except ones dried by SD while the amounts of K and Cu in fresh samples were lower than those in dried samples. The contents of Na and Cu were the highest in dried samples after IRD. The contents of Mg, Ca, Fe and Se were the highest after SD treatment. The contents of K and Zn were the highest after FD, while the level of Mn was higher in samples dried by VD treatment than others. So it can be seen that the mineral contents were influenced by the treatments.

The ash content is an important index of inorganic substances in food. The ash contents in dried samples were in the range of 2.574%~2.874% in comparison with 2.881% in fresh ones. The tangerine peel had high ash content after VD, whereas it had low ash content after HAD. The ash content consistently decreased after drying. Ash content represents the total content of minerals [30]. Similar results were obtained in mineral determination.

Overall all, FD and IRD were the best to preserve soluble solid, reducing sugar, total sugar, crude fiber, and titratable acid for tangerine peels, while SD is better at preserving the minerals in tangerine peel, which is in agreement with previous report on strawberry [31]. However, FD is the most expensive process. The above research findings of this study could provide an empirical proof for quality evaluation and industrialization.

### 3.3. Effect of Different Drying Methods on Total Phenolics Content and Total Flavonoids Content of Tangerine Peels

**Figure 3:**
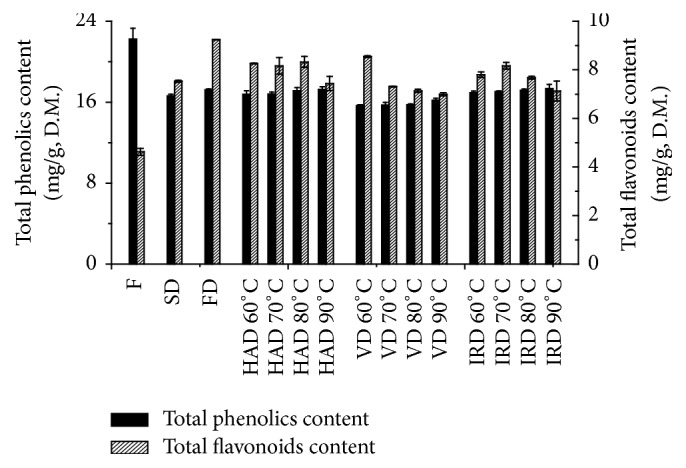
The content variation of total flavonoids content and total phenolics content in tangerine peels dried by different drying methods.

Among the natural functional components, phenolic compounds have attracted wide attention for their promising antioxidant activity and potential roles in the treatment of chronic and degenerative diseases. It was revealed that tangerine peels after drying with different methods presented variable total phenolic contents ranging within 15.66~17.24 mg GAE/g dw (Figure 3), which is lower than the fresh ones (22.24 mg GAE/g dw) (*P* < 0.01). The contents of total phenolic compounds after drying ranked from most to least are IRD/FD/HAD > SD > VD (*P* < 0.05), respectively. With the increase in drying temperatures, a slightly increase in the total phenolics content of tangerine peels was observed in IRD, HAD, and VD. When the plants were dried, the phenolic compounds stored in the plant vacuole are more freely released [32]. IRD could break down the covalent bond of phenolic compounds and liberate low-molecular-weight antioxidants, such as flavonoids and polyphenols. Other heat treatments might destroy the other type of bonds and increased the phenolic content [33, 34].

Flavonoids in tangerine peels (such as hesperidin, naringin, and neohesperidin) are used to treat various physical symptoms such as high cholesterol level, cardiovascular disease, and gastrointestinal function. The total flavonoids contents of tangerine peels dried by 5 drying methods were shown in Figure 3. The total flavonoids content of tangerine peels dried by FD and IRD at 70°C, which was twice of fresh ones, was higher than those dried by the other methods (*P* < 0.05), whereas the lowest content was achieved in tangerine peels after VD at 90°C (6.99 mg GHE/g dw). A considerable amount of total flavonoids contents was also achieved by HAD but was lower than those of FD and IRD at 70°C (*P* < 0.05). Compared with those of fresh ones, the total flavonoids contents of tangerine peels were increased after drying at different temperatures. It was reported that heating and radiation could break down certain types of bonds and release antioxidants such as flavonoids and polyphenols from polymers [33]. Figure 3 also declared that the total flavonoids contents of samples in HAD and IRD were almost not affected by temperature (⩽80°C) but were decreased with further raising temperatures (>80°C). Too high temperature was confirmed to be harmful to preserve flavanone glycosides [35, 36]. It might be related to the fact that naturally occurring antioxidants such as flavonoids might be destroyed by the too high temperature. Therefore, the FD, HAD, and IRD were beneficial in preserving the total flavonoids at low temperatures (<80°C). However, less total flavonoids was liberated with IRD since the drying time was shorter than FD and HAD. In conclusion, the effect of IRD was relatively much better than the other methods used.

### 3.4. Effect of Different Drying Methods on Hesperidin, Synephrine, and Vitamin C Contents of Tangerine Peels

**Figure 4:**
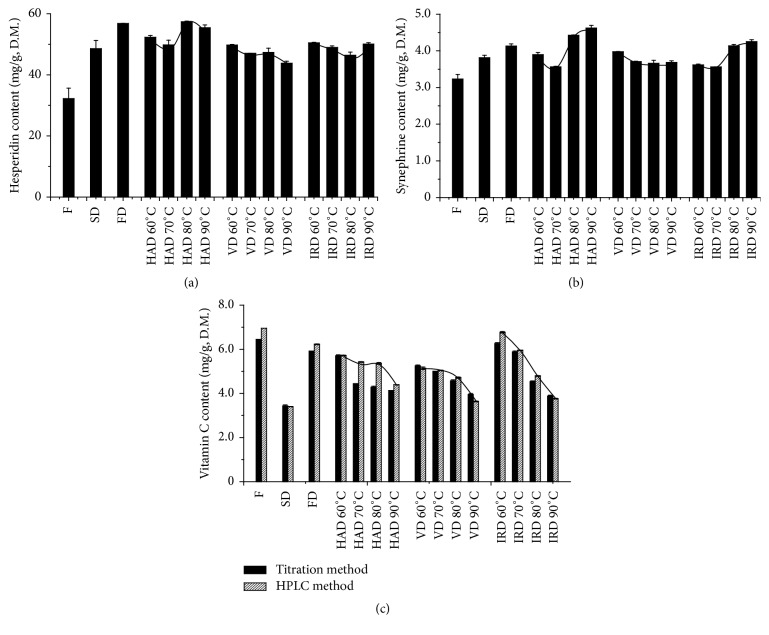
The content variation of hesperidin (a), synephrine (b), and vitamin C (c) in tangerine peels dried by different drying methods.

Hesperidin was the main flavonoid while synephrine was the most important alkaloid in citrus. So the effect of different drying methods was studied on hesperidin and synephrine. As shown in Figure 4(a), the hesperidin content of tangerine peels obtained by FD method was 1.77 times higher than that of fresh ones. In addition, the hesperidin content achieved by this method was also higher than other drying methods. Of all the drying methods, the lowest hesperidin content of samples was obtained by SD. Food processing helps destroy the cell wall and allows phenolic compounds to be released from the insoluble portion of the tangerine peel, which can lead to increased levels of hesperidin content [36]. The HAD was a better drying method to preserve hesperidin than VD and IRD after statistical analysis (*P* < 0.05). FD, HAD, and IRD were all beneficial in preserving hesperidin.

Like hesperidin, the synephrine content in tangerine peels after drying was significantly higher than that in fresh ones (*P* < 0.05) as shown in Figure 4(b). Similarly, it was largely related to the content of essential oils. In fresh peels, the contents of essential oils were high, so the synephrine content was relatively low. After being dried, essential oils in the samples were significantly reduced, and, hence, the proportion of synephrine increased. At high temperatures for VD, the film formed on the surface, which hindered the essential oils' volatilization. In summary, FD, HAD, and IRD were the most suitable to retain synephrine at high temperatures (80°C and 90°C) and dried tangerine peels had much more functions than fresh peels.

The vitamin C content of peels was determined by 2,6-dichloroindophenol titration and HPLC methods (Figure 4(c)). The trends in the results tested by two methods were similar. However, the data obtained by HPLC method was higher than that obtained by titration method. It may be because that 2,6-dichloroindophenol titration method was easily affected by the environment, artificial operation, and the identification of the final point of titration. HPLC method was chosen because of its high accuracy and sensitivity. The vitamin C content of fresh tangerine peels was significantly higher than those of the dried ones (*P* < 0.05). As it is generally known that vitamin C is susceptible to heat and oxygen. Heating and oxidation might accelerate the degradation of vitamin C during the drying process. Therefore, the content of vitamin C decreased gradually along with the increase of temperature [21, 37]. The reason for the low vitamin C content obtained by SD may be the result of samples exposure to air at inconsistent temperatures for a long period. On the other hand, drying at relatively lower temperature and oxygen tension had less effect on the vitamin C content as obtained by FD method compared to other tested methods. Because the samples were dried under short time at low temperature, the peels dried by IRD had significantly higher vitamin C content than those dried by HAD, VD, and SD (*P* < 0.05). Among the drying methods, the IRD was an economical and effective way to preserve vitamin C.

### 3.5. Effect of Different Drying Methods on Antioxidant Activity of Tangerine Peels

**Figure 5:**
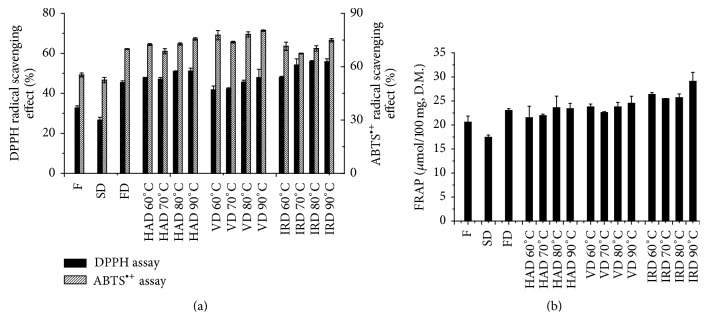
Effect of different methods on the antioxidant activity of tangerine peels: ABTS, DPPH (a), and FRAP (b).

The antioxidant activities of fresh and dried tangerine peels were evaluated by ABTS, DPPH and FRAP assays (Figure 5). The antioxidant activities of the peels after SD were the lowest of the dried peels, which were also lower than the fresh ones (*P* < 0.001). This result was inconsistent with the low contents of vitamin C and phenolic compounds in the peels dried by sunlight. It means the antioxidant activities of dried tangerine peels obtained by traditional methods (SD) had been greatly reduced. The total antioxidant capacity evaluated by ABTS of the peels after VD at 90°C was stronger than others (*P* < 0.05). The peels after IRD had stronger total antioxidant capacity than FD (*P* < 0.05). Similarly, the peels dried by SD and FD had the lowest ability on free radical scavenging (*P* < 0.001) evaluated by DPPH. The ability on free radical scavenging of peels after IRD was stronger than HAD and VD (*P* < 0.05). On reducing ferric, the peels after SD were markedly inferior to other drying methods (*P* < 0.001). Hence, our findings suggested that IRD was a suitable drying method to obtain the high antioxidant activities due to the lower temperatures and shorter drying time necessary for the desired moisture content compared to the other drying methods.

## 4. Conclusions

The impact of the 5 drying methods on nutraceutical components and antioxidant activity of tangerine peels were studied. In general, IRD was an appropriate choice for drying tangerine peels. Compared with the traditional drying method (SD), IRD not only was beneficial in preserving the nutraceutical components of tangerine peels but also shortened the drying time to a greater extent. Altogether, different drying methods may have different advantages to maintain optimal concentrations of the desired components in the dried peels, but the IRD method was found as the most efficient and convenient drying method in preserving main compositions, nutraceutical compositions, and antioxidant activity for tangerine peels. Overall, this study would provide useful information for the preparation method of dried tangerine peel and its industrial applications.

## Figures and Tables

**Table 1:** Effect of different drying methods on main compositions of tangerine peels.

Drying conditions	Soluble solid (%)	Reducing sugar (mg/g dw)	Total sugar (mg/g dw)	Crude fiber (%)	Titratable acid (%)	Ash content (%)	Minerals (ppm)								
							Na	Mg	K	Ca	Fe	Mn	Cu	Zn	Se
F	14.0 ± 0.28^d^	725 ± 4.10^a^	753 ± 41.04^a^	12.8 ± 0.6^a^	0.875 ± 0.064^a^	2.88 ± 0.016^a^	250	1904	6713	11016	22.8	20.2	1.23	14.3	0.014
SD	41.1 ± 1.41^c^	489 ± 8.96^b^	325 ± 26.01^d^	10.8 ± 0.1^b^	0.355 ± 0.049^c^	2.74 ± 0.000^c^	59.3	996	7204	5418	30.2	15.9	1.81	6.10	0.016
FD	51.1 ± 1.41^a^	542 ± 47.84^b^	553 ± 9.92^b^	11.1 ± 0.1^b^	0.670 ± 0.028^b^	2.79 ± 0.001^b^	58.4	805	9605	3934	14.1	6.09	2.07	8.77	0.009
HAD, 80°C	45.3 ± 0.94^bc^	489 ± 94.63^b^	474 ± 4.87^c^	11.0 ± 0.0^b^	0.820 ± 0.028^a^	2.57 ± 0.012^d^	53.4	908	8325	3602	15.3	7.11	1.95	7.32	0.000
VD, 80°C	45.9 ± 2.83^b^	497 ± 85.36^b^	454 ± 8.60^c^	10.5 ± 0.0^b^	0.695 ± 0.049^b^	2.87 ± 0.017^a^	73.2	973	8076	4913	15.5	16.7	1.82	6.55	0.010
IRD, 80°C	54.2 ± 2.83^a^	545 ± 51.44^b^	496 ± 14.26^c^	11.1 ± 0.5^b^	0.835 ± 0.021^a^	2.82 ± 0.033^b^	91.6	795	9452	3936	15.9	13.5	2.09	6.57	0.000

Results are mean ± SD. Different letters in the same column indicate that values are significantly different (*P* < 0.05).
